# Thimble Blistering as a Vehicle for Morphia

**Published:** 1879-08

**Authors:** 


### EDITORIAL, ETC.

*Thimble Blistering as a Vehicle for Morphia.*—J. C. Watson, M. D., of Va., calls attention to a simple method of administering morphia locally, which may prove useful in facial neuralgia where that drug is indicated,—a method which dispenses with the use of the hypodermic syringe. It consists in loosely filling an ordinary sewing thimble with raw cotton and saturating this with strong ammonia water, (ammon. fort.) and pressing the charged thimble on the skin at the spot where it is desired the morphia shall act. In a few minutes a sensation of heat is felt, when the thimble should be removed and the spot gently wiped off to remove any of the ammoniacal liquor. The skin will now be found to have been blistered, and the cuticle can be rubbed off. Dry morphia can now be gently rubbed in, adding a drop of water. This method of blistering is not new, but it furnishes a convenient method of applying morphia and getting rid of the syringe. The blister alone, on the temple or on the gum will be occasionally useful as a counter-irritant in periodontitis, and has the merit of expedition, and is possibly as useful as the irritation produced by cantharides; certainly much less disagreeable.

The writer remembers once promptly relieving a severe periodontitis by making a slight incision over the root of the troublesome tooth, withdrawing the glass barrel of the hypodermic syringe from its metallic case, with the piston in position pushed down as far as it would go, and placing the end of the glass tube over the cut, very slowly withdrawing the piston. Blood followed until the barrel was filled. Great relief resulted. The pain of pressing the tube upon the inflamed surface is the great obstacle in the way of using this "artificial leech," but this might be mitigated by the use of some local anæsthetic. If a longer tube were used, so as to allow the abstraction of more blood, the efficiency should be as great by this method as by natural leeching, to which most patients have an unconquerable aversion.

A very useful local anæsthetic for outward application is made by rubbing together in a mortar equal parts of chloral hydrate and gum camphor. The two solids thus compounded make an oily liquid, which rubbed on the skin is quite effective. Long use of this, or confinement under a bandage or compress will blister very decidedly. H.

